# Comparing the MiniBox™ and the Chestac-8900^®^ for pulmonary function testing

**DOI:** 10.5588/ijtld.23.0212

**Published:** 2023-09-01

**Authors:** T. Matsuki, H. Yanagi, T. Koba, H. Aso, S. Sakaguchi, S. Ito, K. Kouyama, K. Furuta, A. Miyazaki, H. Sumitani, M. Yokoyama, S. Miyamoto, M. Fukai, K. Hashimoto, T. Nii, H. Hashimoto, K. Fukushima, K. Tsujino, K. Miki, H. Kida, A. Kumanogoh

**Affiliations:** 1 Departments of Respiratory Medicine, and; 2 Departments of Clinical Laboratory, National Hospital Organization Osaka Toneyama Medical Center, Toyonaka, Osaka; 3 ASTEM Incorporation, Saga; 4 Department of Respiratory Medicine and Clinical Immunology, Osaka University Graduate School of Medicine, Osaka, Japan

Dear Editor,

The global prevalence of chronic lung diseases is increasing, with a significant number of people affected by chronic obstructive lung disease (COPD), asthma and interstitial lung diseases.^1^–^3^ Patients with long-term COVID-19 may also experience impaired lung diffusion capacity.^4^ Primary care physicians, who have relied primarily on spirometry, need additional parameters to comprehensively evaluate the lung health of their patients.^5^–^8^ This is necessary due to the highly heterogenous nature of each chronic lung disease, with differences in both clinical features and the underlying pathophysiological mechanisms. Although the measurement of static lung volume or diffusion capacity can provide insight into various physiological aspects of lung disease, it often requires expensive and complex equipment (such as whole-body plethysmography and multi-breath helium dilution), which limits their availability in many private clinics.

An alternative tool is the MiniBox™ (PulmOne Advanced Medical Devices, Ra’anana, Israel), which is a table-top plethysmography system that offers a reliable means of measuring spirometry, flow-volume loop, static lung volume and diffusion capacity. It is cost-effective, space-efficient and simple to operate, making it suitable for primary care sites.^9^,^10^ Previous studies have compared the MiniBox system with conventional whole-body plethysmography, but its comparison with helium dilution remains unexplored.^10^,^11^ In Japan, the majority of facilities have adopted the multi-breath helium-dilution method for measuring static lung volume. We therefore performed a single-centre prospective study to compare the Chestac-8900^®^ (Chest, Tokyo, Japan), which employs the multi-breath helium dilution method for measuring static lung volume, with the MiniBox. All subjects provided written informed consent according to a protocol approved by the Institutional Review Board of National Hospital Organization Osaka Toneyama Medical Center, Osaka, Japan (TNH-R-2020048). The study included 60 participants categorised into three groups: 12 healthy volunteers, 24 patients with restrictive lung disease and 24 patients with obstructive lung disease. The baseline clinical characteristics of the participants is shown in Supplementary Table S1. Eleven (45.8%) participants with restrictive and 10 (41.7%) with obstructive lung diseases had chronic respiratory failure and used long-term oxygen therapy.

Spirometric measurements using the MiniBox device were compared with measurements using the Chestac-8900 device. The tidal volume (TV_MB_), expiratory reserve volume (ERV_MB_) and inspiratory reserve volume (IRV_MB_) measured using the MiniBox were not significantly different from the values obtained on the Chestac-8900 (Supplementary Table S2). Similarly, the forced vital capacity (FVC_MB_) and forced expiratory volume in the first second (FEV1_MB_) measured using the MiniBox did not differ significantly from the values obtained using the Chestac-8900 (Supplementary Table S2). In terms of static lung volume measurements, the total lung capacity (TLC_MB_), functional residual capacity (FRC_MB_) and residual volume (RV_MB_) measured using the MiniBox were significantly greater than the values measured by the Chestac-8900 (Supplementary Table S2). A Bland-Altman plot illustrating TLCs of all participants is shown in Figure A. The mean discrepancy was 1.332 L (95% limits of agreement [LoA] –1.113 to 3.778), and this discrepancy was particularly evident in patients with obstructive lung disease (Figure A). To investigate this further, we conducted chest computed tomography (CT) scans in patients with obstructive lung disease to measure TLC and performed morphometric analysis. The TLC measured using CT scans (TLC_CT_) was similar to the TLC measured by the Chestac-8900 (TLC_Chestac_). The mean discrepancy was –0.397 L (95% LoA –2.077 to 1.284) (Figure B). In contrast, the TLC measured using the MiniBox (TLC_MB_) was significantly greater than TLC_CT_ (Figure C). The mean discrepancy was 1.867 L (95% LoA –0.357 to 4.092). The discrepancy between TLC_MB_ and TLC_Chestac_ was negatively correlated with the ratio of FEV1 to FVC (FEV1/FVC) and positively correlated with the percentage of emphysematous lesions on CT (low-attenuation areas [LAA%]) derived from morphometric analysis (Figure D). This suggests that the MiniBox over-estimated TLC compared to the Chestac-8900 and CT, particularly in patients with airflow obstruction and emphysema.

**Figure:**
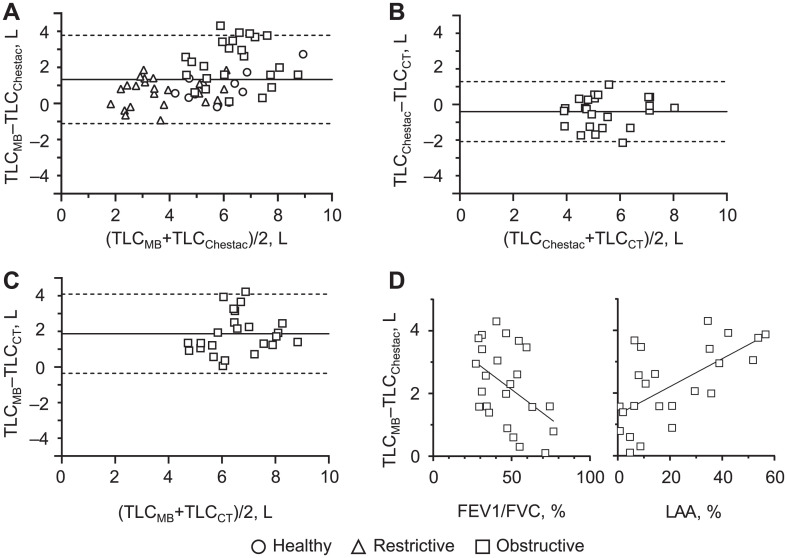
Bland-Altman plots illustrating the comparison of **A)** TLC_MB_ and TLC_Chestac_ for all participants, **B)** TLC_Chestac_ and TLC_CT_ for patients with obstructive disease, and **C)** TLC_MB_ and TLC_CT_ for patients with obstructive disease. **D)** Correlation graphs of FEV1/FVC vs. TLC_MB_ minus TLC_Chestac_ are shown in left, the percentage of the LAA vs. TLC_MB_ minus TLC_Chestac_ on the right. Solid gray lines represent the mean discrepancy; dashed gray lines represent the 95% limits of agreement. TLC = total lung capacity; MB = MiniBox; CT = computed tomography; FEV1 = forced expiratory volume in the first second; LAA = low-attenuation area.

The MiniBox is a compact device with a flow-interruption feature used for measuring lung volumes. It calculates total lung capacity (TLC_MB_) using spirometry data and flow-interruption transients through a machine learning algorithm.^9^,^10^ In this study, TLC_MB_ showed good correlation with TLC measured using both helium dilution (TLC_Chestac_) and chest CT scan (TLC_CT_). However, TLC_MB_ consistently yielded higher values than TLC_Chestac_ or TLC_CT_, particularly in patients with obstructive lung disease. For the MiniBox, the predictive equation for static lung volume was generated from data based on the original set of individuals, whose static lung volumes (measured using plethysmography) served as the reference.^12^ Although this study focused on an Asian population, the results were consistent with previous studies, suggesting that race-related differences may be minimal. However, the study had limitations such as being conducted at a single centre with a relatively small sample size, highlighting the need for future multicentre validation studies targeting different disease populations.

In terms of diffusion capacity measurements, there were good correlations between the MiniBox and Chestac-8900 for alveolar volume (VA), carbon monoxide transfer coefficient (K_CO_) and diffusion capacity of the lungs for carbon monoxide (DL_CO_) (Supplementary Figure S1). However, VA measured using the MiniBox was significantly larger than VA measured by the Chestac-8900. On the other hand, the K_CO_ measured using the MiniBox was significantly smaller than the K_CO_ measured by the Chestac-8900. These differences offset each other, resulting in equivalent DL_CO_ values for both devices (Supplementary Table S2). The Minibox uses methane (which has a higher solubility in blood and lungs) as the tracer gas instead of helium as used in the Chestac-8900, which might have contributed to the observed difference in VA and Kco values between the Minibox and the Chestac-8900.

Currently, whole-body plethysmography is the gold standard for measuring lung volumes, but each method has limitations.^13^ MiniBox’s advantage lies in its ability to measure static lung volumes during normal breathing without the need for inert gas inhalation, which allows for repeated measurements with improved day-to-day repeatability compared to other methods. This makes it suitable for longitudinal monitoring and management of patients, especially those with COPD.^6^,^14^,^15^
