# Supplementary material for: Comparing the MiniBox™ and the Chestac-8900® for pulmonary function testing
Source: Int J Tuberc Lung Dis. 2023 Sep 1;27(9):709–11. doi: 10.5588/ijtld.23.0212 (PMC10443784; doi:10.5588/ijtld.23.0212)

http://dx.doi.org/10.5588/ijtld.23.0212

Comparing the MiniBox™ and the Chestac-8900 for pulmonary function testing

Supplementary Table S1. Baseline clinical characteristics of the participants.

	Total (n=60)	Healthy (n=12)	Restrictive (n=24)	Obstructive (n=24)	P-value
Male	40 (66.6)	6 (50.0)	15 (62.5)	19 (79.1)	P=0.185
Age, years	72 (59-79)	34 (25-50)	75 (68-81)	75 (71-79)	P<0.001
Height, cm	162 (158-168)	164 (158-168)	160 (155-166)	162 (159-170)	P=0.377
Weight, kg	58 (49-64)	57 (49-64)	57 (51-62)	58 (49-66)	P=0.979
BMI	22 (19-24)	21 (20-22)	22 (20-24)	21 (19-24)	P=0.757
Smoking, pack-years	1.4 (0-2.3)	0 (0-6.9)	16 (0-32)	47 (31-69)	P<0.001
LTOT	21 (18.3)	0	11 (45.8)	10 (41.7)	P<0.001

Abbreviations: restrictive, restrictive lung disease; obstructive, obstructive lung disease; BMI, body mass index; LTOT, long-term oxygen therapy. Data are shown as n (%) or the median (interquartile range).

Supplementary Table S2. Comparison between the Chestac-8900 and the MiniBox

n=60	Chestac-8900	MiniBox	P-value
FVC, L	2.76 (1.79-3.61)	2.86 (1.86-3.77)	P=0.616
Healthy (n=12)	3.56 (2.94-4.76)	3.81 (3.02-5.04)	P=0.698
Restrictive (n=24)	1.75 (1.20-3.04)	1.80 (1.23-3.16)	P=0.761
Obstructive (n=24)	3.05 (2.05-3.68)	3.12 (2.18-3.71	P=0.612
FEV_1, L	1.69 (1.08-2.60)	1.70 (1.01-2.60)	P=0.852
Healthy (n=12)	3.12 (2.55-3.85)	3.23 (2.56-3.93)	P=0.852
Restrictive (n=24)	1.59 (1.12-2.29)	1.56 (1.11-2.33)	P=0.779
Obstructive (n=24)	1.31 (0.74-1.84)	1.35 (0.78-1.84)	P=0.890
FEV_1/FVC, %	75.6 (50.7-86.4)	73.7 (51.1-85.3)	P=0.691
Healthy (n=12)	84.2 (81.1-86.4)	82.4 (78.4-85.3)	P=0.647
Restrictive (n=24)	86.4 (79.4-95.8)	85.6 (75.9-94.2)	P=0.739
Obstructive (n=24)	46.5 (32.9-54.7)	44.5 (31.8-52.4)	P=0.688
TV, L	0.71(0.59-0.88)	0.67(0.51-0.84)	P=0.439
Healthy (n=12)	0.66 (0.54-0.73)	0.77 (0.55-0.87)	P=0.317
Restrictive (n=24)	0.66 (0.49-0.82)	0.55 (0.45-0.77)	P=0.807
Obstructive (n=24)	0.81 (0.69-1.07)	0.70 (0.60-0.85)	P=0.187
ERV, L	0.81(0.50-1.23)	0.99(0.64-1.38)	P=0.249
Healthy (n=12)	1.37 (1.07-1.77)	1.71 (1.42-1.89)	P=0.292
Restrictive (n=24)	0.50 (0.36-0.76)	0.63 (0.42-1.18)	P=0.181
Obstructive (n=24)	0.91 (0.59-1.15)	0.98 (0.76-1.22)	P=0.493
IRV, L	1.12(0.55-1.59)	0.93(0.58-1.70)	P=0.880
Healthy (n=12)	1.67 (1.24-2.38)	1.73 (0.97-2.19)	P=0.808
Restrictive (n=24)	0.60 (0.28-0.92)	0.57 (0.26-1.06)	P=0.999
Obstructive (n=24)	1.15 (0.61-1.45)	1.36 (0.70-1.71)	P=0.283
FRC, L	2.79 (1.96-3.18)	3.85 (2.8.5-5.09)	P<0.001
Healthy (n=12)	2.96 (2.56-3.35)	4.00 (3.32-4.39)	P=0.022
Restrictive (n=24)	1.78 (1.42-2.66)	2.62 (1.97-3.24)	P=0.016
Obstructive (n=24)	3.12 (2.69-3.59)	5.61 (4.75-6.48)	P<0.001
RV, L	1.58 (1.26-2.25)	2.37 (1.96-3.95)	P<0.001
Healthy (n=12)	1.46 (1.26-1.82)	2.04 (1.89-2.57)	P=0.037
Restrictive (n=24)	1.31 (1.03-1.54)	1.98 (1.41-2.23)	P=0.005
Obstructive (n=24)	2.26 (1.61-2.80)	4.48 (3.59-5.21)	P<0.001
TLC, L	4.63 (3.51-5.64)	5.74 (4.08-7.68)	P<0.001
Healthy (n=12)	5.85 (4.66-6.04)	5.74 (5.37-6.98)	P=0.157

Restrictive (n=24)	2.86 (2.35-4.57)	3.78 (3.14-5.38)	P=0.107
Obstructive (n=24)	4.99 (4.51-5.84)	7.88 (6.21-8.53)	P<0.001
DL_{CO}, mL/min/mmHg	9.7 (5.1-15.7)	9.4 (6.4-13.8)	P=0.942
Healthy (n=12)	22.1(19.1-25.9)	20.7(20.4-22.2)	P=0.444
Restrictive (n=24)	6.0(3.9-9.5)	7.2(6.1-8.7)	P=0.347
Obstructive (n=24)	9.9(5.0-13.4)	9.4(6.3-11.2)	P=0.627
VA, L	3.32 (2.43-4.17)	4.67(3.19-5.39)	P<0.001
Healthy (n=12)	4.38(3.53-4.84)	5.39(4.71-6.28)	P=0.008
Restrictive (n=24)	2.34(1.79-3.54)	3.19(2.70-4.96)	P=0.004
Obstructive	3.65(2.79-4.26)	4.79(3.51-5.63)	P=0.009
K_{CO}/VA, mL/min/mmHg/L	2.93(1.80-4.83)	2.60(1.53-3.42)	P=0.011
Healthy (n=12)	5.24(5.02-5.62)	4.02(3.59-4.33)	P<0.001
Restrictive (n=24)	2.58(1.50-4.03)	2.64(1.56-3.14)	P=0.264
Obstructive (n=24)	2.38(1.69-3.34)	1.95(1.33-2.48)	P=0.062

Abbreviations: FVC, forced vital capacity; FEV_1, forced expiratory volume in the first second; TV, tidal volume; ERV, expiratory reserve volume; IRV, inspiratory reserve volume; FRC, functional residual capacity; RV, residual volume; TLC, total lung capacity; DL_{CO}, diffusion capacity of the lungs for carbon monoxide; VA, alveolar volume; K_{CO}, carbon monoxide transfer coefficient. Data are shown as the median (interquartile range).

Supplementary Figure S1.

Correlation graph of MiniBox-derived diffusion capacity of the lungs for carbon monoxide (DLCO-MB) vs Chestac-8900-derived DLCO (DLCO-Chest) (A). MiniBox-derived alveolar volume (VAMB) vs Chestac-8900-derived VA (VAChest) (B). MiniBox-derived KCO (KCO-MB) vs Chestac-8900-derived KCO (KCO-chest) (C). The gray dashed line is the identity line.

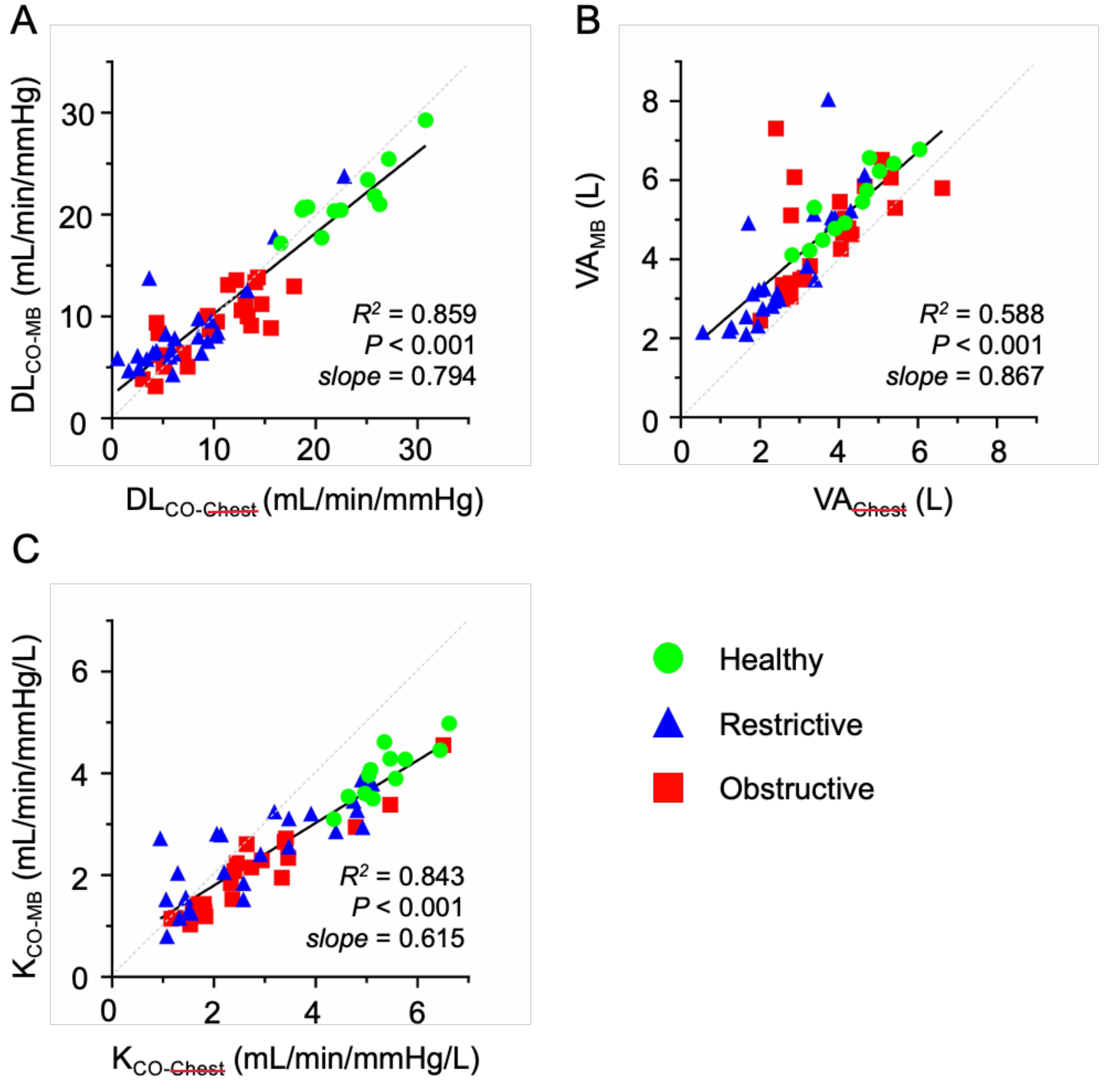

Supplement: Supplementary file 1 [file iutld_ijtld_23.0212_supplementarydata1.pdf]
